# Role of Transcription Factor Fli-1 in Inflammation and Autoimmune Diseases

**DOI:** 10.3390/biom15040480

**Published:** 2025-03-25

**Authors:** Xuan Wang, Xian K. Zhang

**Affiliations:** 1 Department of General Practice, Xiangya Hospital Central South University, and International Joint Research Center for Medical Metabolomics of Xiangya Hospital, Changsha 410008, China; 2 Department of Medicine, Division of Rheumatology & Immunology, Medical University of South Carolina, Charleston, SC 29403, USA

**Keywords:** Fli-1, inflammatory mediator, transcriptional factor, cytokine, chemokine, autoimmune disease

## Abstract

Friend leukemia virus integration 1 (Fli-1), a member of the ETS family of transcription factors, plays an essential role in diverse biological processes. Recent studies have underscored the significance of Fli-1 in modulating inflammation and autoimmune diseases via the regulation of inflammatory responses. Specifically, Fli-1 exerts control over inflammatory processes, influencing key effectors and signaling pathways associated with conditions such as systemic lupus erythematosus, scleroderma, cancer, and sepsis. This review aims to summarize the emerging roles of Fli-1 in inflammation and autoimmune diseases, with a focus on elucidating the underlying molecular mechanisms and exploring the potential therapeutic implications.

## 1. Introduction

With the continuous advancement of natural science, academics have suggested that it is time to redefine inflammation, challenging previous definitions. Rudolph Virchow, the father of modern pathology, identified four types of inflammation—exudative, infiltrative, parenchymatous, and proliferative—and emphasized the importance of the inflammatory stimulus [1]. In essence, inflammation has been redefined as the innate immune response to potentially harmful stimuli such as pathogens, injury, and metabolic stress [1,2]. As the understanding of inflammation deepens, scientists have gained extensive knowledge regarding inflammatory signaling pathways (classical and non-classical) that are linked to diverse mediators. The core function of inflammation is to restore homeostasis by responding to harmful stimuli via mediators and immune cell migration. Hence, regulating these pathways to control mediators and cell migration is critical for managing related diseases.

Friend leukemia virus integration 1 (Fli-1) was first identified in 1990 by Ben-David et al. as a common proviral integration site [3]. Fli-1 is a transcription factor that belongs to ETS gene family and traditionally binds to the short core consensus DNA binding motif GGAA/T through a winged helix-turn-helix domain [4]. Both human and murine Fli-1 consist of 452 amino acids (aa) and contain various domains: 5’ ETS domain (aa 121–196), FLS domain (Fli-1 specific domain, aa 205–292), 3′ ETS domain (aa 277–360), and CTA domain (carboxy-terminal transcriptional activation domain, 402–452) [5,6]. The FLS and 5′ ets domains are generally referred to as ATA (amino terminal transcriptional activation) domains. Thereinto, ATA and CTA domains contribute to the transcriptional activity of the Fli-1 protein and the 3′ ETS domain is found to be responsible for sequence-specific DNA-binding activity. Fli-1 regulates the expression of multiple target genes related to proliferation, apoptosis, angiogenesis, differentiation, development, and the immune response [4,7]. Importantly, a growing body of evidence from our laboratory and others links the aberrant expression of Fli-1 to the activation and regulation of various inflammation-related genes [8,9,10,11,12,13,14]. Fli-1/inflammatory mediator axis has been shown to be involved in the pathogenesis of various inflammatory diseases including systemic lupus erythematosus (SLE), systemic sclerosis (SSc), sepsis, and cancer [11,15,16,17,18,19].

## 2. Role of Fli-1 in Regulating Inflammation

### 2.1. Inflammatory Mediators Regulated by Fli-1

**Figure 1:**
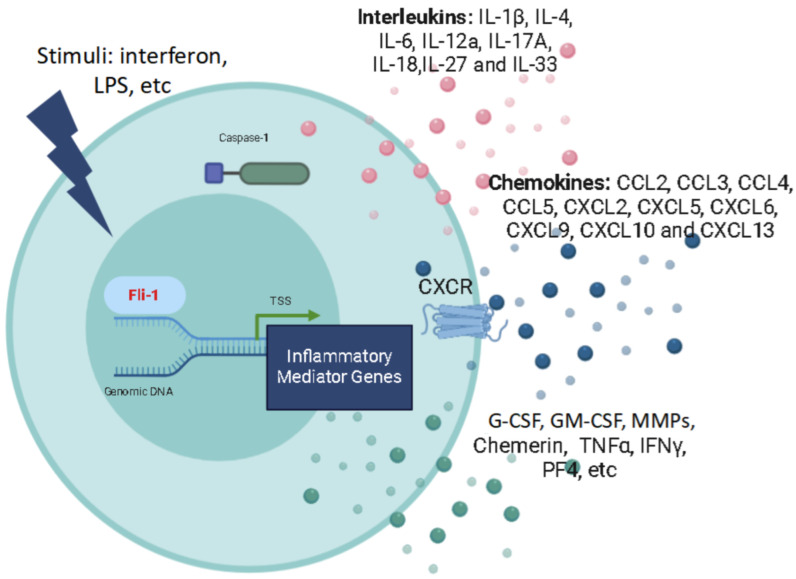
The impact of aberrant Fli-1 on the regulation of inflammatory mediators: Aberrant expression or activation of Fli-1 due to various stimuli result in the dysregulation of interleukins, chemokines, and other inflammatory factors. The activation and secretion of these inflammatory mediators are critical in driving various inflammatory responses.

Recently, evidence has demonstrated that Fli-1 serves as a critical regulator of multiple inflammatory mediators, including interleukins (ILs), chemokines, and colony-stimulating factors. This involvement in the control of diverse inflammatory processes underscores Fli-1′s pivotal role in inflammation, as illustrated in Figure 1.

Various interleukins have garnered significant attention as potential effectors in the pathology and physiology of inflammatory diseases. Studies in Fli-1 knock-down mice revealed the exact effects of Fli-1 on IL-1β, IL-4, IL-6, IL-10, IL-12a, IL-17A, IL-18, and IL-33 in different disease models (Table 1). In Fli-1 heterozygote MRL/lpr lupus mice, the expressions of IL-1β, IL-6, IL-17A, and IL-18 in the kidney were decreased; the levels of IL-4 in splenic T cells and IL-6 in both serum and splenic T cells were also reduced [11,14,20,21]. However, IL-12a transcripts were upregulated in the B cells of Fli-1 heterozygote MRL/lpr mice [22]. Fli-1′s regulation of the IL-1β and IL-18 genes in lupus mice was further confirmed in the lung pericytes of Cecal ligation and puncture (CLP)-induced sepsis models [19]. Nevertheless, the regulatory effect of Fli-1 on expression of interleukins including IL-1β, IL-4, IL-6, IL-10, IL-12a, IL-17A, and IL-33 is the opposite in skin samples from a bleomycin-induced SSc murine model [23,24]. Therefore, the regulation of Fli-1 on inflammatory mediators varies from disease to disease, which needs further investigation.

In vitro, knockdown of Fli-1 decreased LPS-induced IL-6 expression in MS1 endothelial cells and lung pericytes, reduced IL-10 expression in primary human monocytes after exposure to LPS with or without IFNγ, attenuated bacterial outer membrane vesicles (OMV, contain LPS), and induced IL-18 expression in mouse lung pericytes and IL-27 expression in LPS-induced in mouse peritoneal macrophages [11,20,25,26]. Taken together, the inhibition of Fli-1 expression in vitro can reduce the pro-inflammatory interleukin production induced by LPS or other stimuli.

Chemokines are a family of low-molecular-weight proteins that induce the chemotaxis of inflammatory cells, which play a crucial role in the process of inflammatory diseases [27]. As key orchestrators of leukocyte trafficking in injured areas during inflammation, immune surveillance, and cancer progression, chemokines and their receptors represent important pharmacologic targets for therapeutic intervention [28]. Chemokines bind to G protein-coupled receptors (GPCRs) that are based on the conserved N-terminal cysteine residues in their mature ligands [29]. Fli-1 has a regulatory effect on a variety of chemokine ligands, including CCL2 (MCP-1, C-C motif ligand 2), CCL3, CCL4, CCL5 (RANTES), CXCL2 (C-X-C motif ligand 2), CXCL5, CXCL6, CXCL9, CXCL10, and CXCL13 (Table 2). Previous studies have shown that Fli-1 is a positive regulator of CCL2, CCL3, CCL4, CCL5, CXCL9, and CXCL10 in the kidneys of Fli-1 heterozygote knockout mice with lupus and CXCL5 in dermal small vessels from Fli-1 knockout mice [19,30,31,32]. However, Fli-1 expression and activation were also reported to down-regulate CCL2 expression in skin samples derived from a bleomycin-induced SSc model [23]. Meanwhile, whether Fli-1 regulates the expression of CXCL2, CXCL6, CXCL13, and other chemokines in animal models remains unclear. Thus, the expression and activation of Fli-1 are positively correlated with the expression of most chemokines.

As for regulation of chemokines in vitro, inhibition of Fli-1 decreased the production of CCL2, CCL5, CXCL2, and CXCL5 in various types of endothelial cells and reduced CXCL6 expression in mouse peritoneal macrophages [10,13,32,33]. However, knockdown of Fli-1 increased LPS-induced CXCL6 production in human dermal fibroblasts and human dermal microvascular endothelial cells (HDMECs) and enhanced LPS-induced CXCL13 production in mouse peritoneal macrophages [33,35].

In addition to regulating IL and chemokine gene expression, Fli-1 also functions as a transcriptional factor for other inflammation-related genes, including granulocyte colony stimulating factor (G-CSF), granulocyte-macrophage colony stimulating factor (GM-CSF), matrix metalloproteinases (MMPs), caspases, and platelet factor 4 (PF4) (Table 3). Further investigation of the role and mechanisms of Fli-1 in these inflammatory mediators in various cell types and animal models is needed.

### 2.2. Fli-1 Affects Cellular Processes

To study how the expression of Fli-1 affects the infiltration of inflammatory cells into inflammatory sites, we generated transgenic enhanced green fluorescent protein (GFP) transgenic MRL/lpr mice. Inflammatory cells from wild-type MRL/lpr mice showed significantly increased infiltration into the kidneys compared to cells from Fli-1 heterozygote knockout MRL/lpr mice. The chemotaxis of inflammatory cells from Fli-1 heterozygote knockout MRL/lpr mice towards each chemokine was significantly decreased compared to inflammatory cells from wild-type MRL/lpr mice in the Transwell migration assay in vitro [30]. Additionally, T cells with reduced expression of Fli-1 showed decreased migration into the inflamed kidney in MRL/lpr mice compared to wild-type T cells [31].

A recent publication demonstrates that Fli-1 dynamically regulates various T cell subsets involved in allogeneic responses and the development of acute graft-versus-host disease (aGVHD) and chronic GVHD (cGVHD). Notably, T cells with heterozygous Fli-1 deficiency induced the mildest form of GVHD, as shown by reduced Th1 and Th17 cell differentiation, compared to T cells with homozygous Fli-1 deficiency or wild-type T cells. Single-cell RNA sequencing revealed that Fli-1 distinctly modulates CD4+ and CD8+ T cell responses, promoting the transcription of Th1/Th17 pathways and T-cell-receptor-inducible transcription factors in CD4+ T cells, while inhibiting activation- and function-related gene pathways in CD8+ T cells. Importantly, low doses of camptothecin, topotecan, or etoposide significantly reduced GVHD severity by acting as potent Fli-1 inhibitors without compromising the graft-versus-leukemia (GVL) effect. This finding was further supported in a xenograft model, where GVHD was induced by human T cells [41]. B cells with reduced expression of Fli-1 have significantly decreased proliferation compared to wild-type B cells upon stimulation of the BCR [22]. Reducing Fli-1 expression in MRL/lpr and NZBW mice significantly decreased antibody production [42].

Studies have shown that Fli-1 is mainly localized in the nucleoplasm and nuclear bodies [43]. As for the tissue catalogue, the human Fli-1 gene has been found in brain, skin, myeloid, lymphoid, lung, abdominal, breast, reproductive system, kidney, urinary, sarcoma, fibroblast, endothelium, and miscellaneous tissues, etc. According to The Human Protein Atlas, human Fli-1 was highly expressed in HELs (human erythroleukemia cells), HUVECs (endothelial cells originating in umbilical veins), REHs (cancer cell lines originating in lymphoid), and TIMEs (endothelial cell lines originating in skin). Thus, Fli-1 participates in the inflammation associate with myelocytes, lymphocytes, and endothelial cells on the basis of its expression pattern. Fli-1 is expressed in hematopoietic and endothelial cells even under normal physiological conditions [44]. Emerging evidence has demonstrated that inhibition of Fli-1 in various endothelial cells, macrophages, and splenic T cells significantly reduced the great mass of pro-inflammatory ILs and chemokine production (Table 1 and Table 2); however, in skin-derived cells, Fli-1 inhibition did not reduce and may instead increase the expression of inflammatory factors.

For murine cells, the expression pattern of Fli-1 is different from human cells. Although human monocytes and mesangial cells have been shown have a degree of Fli-1 expression, Fli-1 expression in mouse monocytes and mesangial cells has not been detected. Fli-1 is closely related to embryonic and organ development, and its expression varies from species to species. The regulation of Fli-1 in inflammatory mediators has been confirmed in various mouse cell types, including endothelial cells, pericytes, and fibroblasts. The regulatory role of Fli-1 in inflammatory mediators is consistent across species.

Microglial cells, which represent approximately 10% of brain cells, are recognized as the resident immune cells of the central nervous system [45]. We have found that Fli-1 is highly expressed in microglial cells and that the expression of many inflammatory cytokines was significantly reduced in microglial cells with reduced Fli-1 expression compared to the wild-type microglial cells following stimulation with interferon.

### 2.3. Mechanisms Underlying Fli-1 Regulation in Inflammatory Mediators

To explore the possible mechanisms by which transcription factor Fli-1 intrinsically regulates inflammatory mediators, previous studies have primarily analyzed whether Fli-1 directly binds to the promoter of inflammation-related genes. It has been reported that Fli-1 directly binds to the promoter of IL-6, IL-27, CCL2, CCL5, CXCL2, G-CSF, caspase-1, and PF4, etc., as evidenced via CHIP assay. Moreover, Fli-1 also drives transcription from the promoter of IL-6, IL-27, CCL2, CCL5, CXCL2, CXCL10, G-CSF, and caspase-1 as measured using a luciferase assay [9,10,11,12,13,19,26,34,38]. These results strongly suggest that directly binding to the promoter is an important mechanism behind Fli-1 regulation of these inflammatory mediators. To further elucidate the mechanisms behind the activation of G-CSF by Fli-1, Mara L. et al. demonstrated that deletion of the distal region of the G-CSF promoter resulted in a 43% loss of activity, indicating that factors other than Fli-1 regulate transcription within this portion of the promoter [12].

The modification of Fli-1 is likely involved in its activation and its ability to bind to target promoters, thereby influencing gene expression. Post-translational modifications such as acetylation and phosphorylation play critical roles in regulating Fli-1′s transcriptional activity. Mutation of the acetylation site leads to a significant increase in Fli-1 activation of the G-CSF promoter, suggesting that acetylation at this site negatively regulates Fli-1 function [12]. This finding is further supported by studies showing that co-transfection of Fli-1 with histone acetyltransferases (HATs), such as p300 or PCAF (p300/CBP-associated factor), results in a statistically significant decrease in G-CSF promoter activation. This suggests that acetylation may destabilize Fli-1 or interfere with its ability to recruit the essential co-activators needed for full transcriptional activity [46]. In addition to acetylation, phosphorylation also modulates Fli-1 activity. For example, Protein Kinase C Delta (PKCδ) phosphorylates Fli-1 at threonine 312, which has been linked to changes in transcriptional regulation. A phosphorylation-deficient mutant of Fli-1 was found to exhibit an increased inhibitory effect on the COL1A2 gene, indicating that phosphorylation at this site reduces Fli-1′s ability to repress certain target genes [47]. We also reported that the phosphorylation of Fli-1 affected the expression of GM-CSF [36].

Another key mechanism of Fli-1 activation is its interaction with other transcription factors, which can enhance or repress gene expression depending on the context. For example, Fli-1 interacts with the p65 subunit of NF-κB, leading to the synergistic enhancement of CCL2 transcription. CCL2 is a critical chemokine involved in immune cell recruitment during inflammation, and the cooperative interaction between Fli-1 and p65 suggests that Fli-1 plays an essential role in inflammatory gene regulation [8]. Indeed, Fli-1 increases IL-27 p28 promoter-controlled gene transcription and cooperates with IRF1 (Interferon regulatory factor 1) to regulate IL-27 p28 gene expression [26]. Fli-1 interacts with the Ets-1 transcription factor to drive transcription from the CCL2 promoter. Meanwhile, Fli-1 and NFκB p65 enhance transcription from the CCL2 promoter, while NFκB p50 and Sp1 (Specificity protein 1) suppress this process [9]. Furthermore, Ets1 (E26 transformation-specific sequence-1) acts as a dominant negative transcription factor to Fli-1 in the context of the CCL5 promoter [10]. In the regulation of CXCL2, NFκB has been shown to act in an additive manner with Fli-1 [13]. These studies further revealed that indirect protein–protein interactions, especially Fli-1 co-working with other transcription factors such as Ets1, NFκB, Sp1, IRF1, etc, also contribute to the regulation of inflammation-related genes.

**Figure 2:**
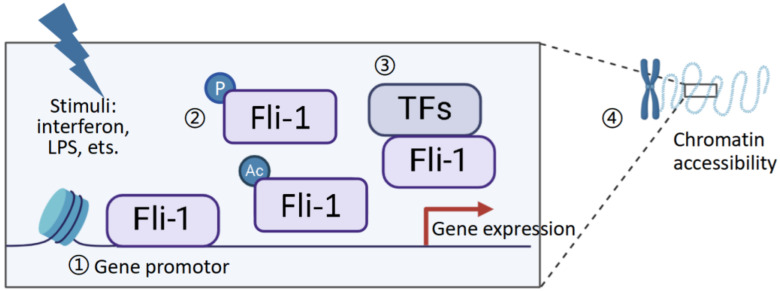
Mechanism of Fli-1 involvement in the transcriptional regulation of inflammation-related genes: A variety of stimulants promote the transcriptional activation of Fli-1 through the following mechanisms: ① In the nucleus, transcription factor Fli-1 directly binds to the promoter regions of inflammation-related genes, driving the transcription of inflammatory mediators such as IL-6, IL-27, CCL2, CCL5, CXCL2, G-CSF, and caspase-1. ② Post-translational modifications of Fli-1, including acetylation and phosphorylation, enhance its transcriptional activity. ③ Also, protein–protein interactions, particularly the collaboration of Fli-1 with other transcription factors such as Ets1, NFκB, Sp1, and IRF1 etc, also play a critical role in the transcriptional activation of Fli-1. ④ In addition, Fli-1 also participates in influencing chromatin accessibility, thereby regulating gene transcription. Fli1 genomic binding was related to changes in chromatin accessibility and TEFF biology.

Taken together, these findings highlight the importance of post-translational modifications and transcription factor interactions in fine-tuning Fli-1 activity, allowing it to regulate diverse biological processes, including the immune response, hematopoiesis, and tumorigenesis. Also, intriguingly, Fli-1 genomic binding was associated with changes in chromatin accessibility and effector T cell biology. The role of Fli-1 in regulating chromatin accessibility remains novel and warrants further investigation in future studies [48] (Figure 2).

## 3. Implications of Fli-1 in Autoimmune/Inflammatory Diseases

### 3.1. The Role of Fli-1 in Lupus

Systemic lupus erythematosus is the most common autoimmune inflammatory disease; it causes widespread inflammation and tissue damage in the multiple affected organs. SLE patients exhibit an increased expression of Fli-1 in peripheral blood lymphocytes that is positively correlated with the clinical disease activity [49]. This implies that Fli-1 may play a potential role in SLE patients. Moreover, the inhibition of Fli-1 in two lupus mouse strains (MRL/lpr and NZM2410) significantly attenuated lupus disease severity, as evidenced by prolonged survival [42,50], decreased pathological damage and infiltrating inflammatory cells in kidney [30], reduced total B cell and activated B cell populations in the spleens and autoantibody production [22], and lessened pathogenicity of T cells with TCR-specific activation and glycosphingolipid levels [21,31].

**Figure 3:**
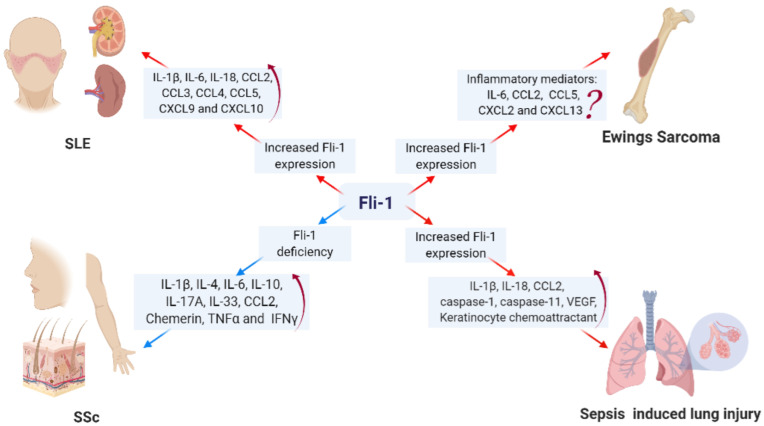
Implications of Fli-1 in inflammation-related diseases through regulation of inflammatory factors: The expression of Fli-1 is upregulated in the active state of SLE, cancer, and sepsis, while it is reduced in SSc. Suppression of Fli-1 in animal models of lupus and sepsis resulted in decreased production of inflammatory mediators, suggesting a role for Fli-1 in promoting inflammation during these conditions. Conversely, inhibition of Fli-1 exacerbated disease progression and increased the secretion of inflammatory mediators in the skin of SSc models, indicating that Fli-1 may play a protective role in this context. In tumor models, the expression of various inflammatory mediators is upregulated; however, whether Fli-1 plays a pivotal role in regulating these mediators remains to be fully elucidated and warrants further investigation.

Fli-1′s role in lupus may be partly due to the production of pro-inflammatory mediators such as IL-6 and CCL2, which have been alleviated in endothelial cells and splenic T cells in Fli-1 heterozygous lupus mice [11,14,19,30]. Moreover, multiple inflammatory mediators including IL-1β, IL-6, IL-18, CCL2, CCL3, CCL4, CCL5, CXCL9, and CXCL10 were reduced in the kidneys of Fli-1 heterozygote knockout mice with lupus [11,14,20,30,31] (Figure 3). These mediators are involved in the recruitment and function of inflammatory cells within injured tissues, such as kidneys. Therefore, Fli-1 affects lupus development by directly regulating the expression of inflammatory mediators and the migration of inflammatory cells.

### 3.2. Fli-1 in SSc

Systemic sclerosis is another autoimmune inflammatory disease characterized by fibrosis of the skin (especially the face and both upper limbs) and internal organs due to increased collagen production. Unlike lupus, expression of Fli-1 was greatly reduced in endothelial and peri-endothelial cells in skin from SSc patients [51]. Also, Fli-1 gene expression is suppressed at the transcriptional level by an epigenetic mechanism in SSc fibroblasts [52]. Fli-1-deficient mice developed SSc by developing an SSc-like phenotype in dermal fibroblasts, endothelial cells, and macrophages [23]. Reduced expression of Fli-1 led to upregulation of fibrogenic genes (collagen I, αSMA), downregulation of MMP-1 in fibroblasts, and the loss of pericytes and vasculopathy in skin [53]. Matrix Metalloproteinases are a group of proteolytic enzymes responsible for degrading the extracellular matrix. In fibrosis, there is an increase in extracellular matrix accumulation when MMP1 levels are reduced [37]. Consequently, Fli-1 deficiency was identified as a predisposing factor for SSc (Figure 3).

In addition, numerous studies have confirmed that inflammatory mediators including IL-1β, IL-4, IL-6, IL-10, IL-17A, IL-33, CCL2, Chemerin, TNFα, and IFNγ were enhanced in skins from SSc mice when Fli-1 was knocked down [23,24,25,39,53]. Th2/Th17-polarized inflammation and increased inflammatory cell (macrophages and mast cells) infiltration has been confirmed in the skins of bleomycin-treated Fli-1 heterozygote mice [23]. Different from lupus, Fli-1 has an inverse correlation with the production of inflammatory mediators in skin. As such, Fli-1 plays different roles in diverse tissues and animal models, which likewise suggests that this transcription factor is crucial for homeostasis.

### 3.3. Fli-1 in Cancer

Cancer remains one of the most intensely studied fields in biomedical research [54]. Most attention about Fli-1 in cancer has been paid to Ewing’s sarcoma and hematologic malignancies [55,56]. The malignant transformation and blood cell lineage development mediated by Fli-1 have been focused on as the underlying mechanisms in many tumor studies. Actually, malignant neoplasms often resemble inflamed tissues, with immune cells regulating one another through surface molecules or secreted mediators [57,58]. Increasing evidence had suggested that local immune responses and systemic inflammation substantially contribute to the development and progression of malignancies [58]. Inflammatory cytokines and chemokines play a critical role in tumor progression through promoting angiogenesis, metastasis, the subversion of adaptive immunity, and changing the response to hormones and chemotherapeutic agents [59]. For example, the cytokine IL-6 was upregulated in various types of cancer and a deficiency of IL-6 rendered mice resistant to the development of murine plasmocytoma [59,60]. Chemokines such as CCL2, CCL5, CXCL2, and CXCL13 have been detected in neoplastic tissues as products of tumor cells [61]. Since Fli-1 is a key regulator of various cytokines and chemokines, including IL-6, CCL2, CCL5, CXCL2, and CXCL13, the role of Fli-1 in promoting cancer development may partly be due to its role in driving the expression of these inflammatory mediators. Thus, the beneficial effect of inhibiting Fli-1 in cancer cells may, at least in part, be attributed to decreased inflammation. Chen, Z et al. demonstrated that reduced Fli-1 in CD8+ T cells enhances protection against infections and tumors in mice by preventing exhaustion and boosting effector function; Fli-1 regulates gene expression by modulating chromatin accessibility, thereby enhancing protective immunity against infections and cancer [48].

### 3.4. Fli-1 in Sepsis

Sepsis refers to the life-threatening organ dysfunction caused by a dysregulated host response to infections [62]. Li, P. et al. demonstrated that Fli-1 expression was upregulated in lung pericytes from CLP-induced septic mice in vivo and in LPS-stimulated lung pericytes in vitro. Disrupted Fli-1 expression inhibited LPS-induced inflammatory ILs (IL-1β and IL-18) and a chemokine (CCL2) in cultured lung pericytes, blocked OMVs-induced caspase-1 and caspase-11 expression, and prevented CLP-induced pericyte loss, vascular leak, and improved survival [19,20] (Figure 3). Through the modulation of NFκB signaling and regulated by miR-145a, Fli-1 has been confirmed to be involved in the regulation of sepsis-associated microvascular dysfunction and organ injury [63]. Sepsis is characterized by an overwhelming inflammatory response, which is driven, in part, by enhanced cytokine and chemokine production. Therefore, Fli-1 may contribute to the pathogenesis of sepsis and represents a novel therapeutic target due to its direct impact on transcription of inflammatory mediators.

## 4. Fli-1 in Pharmaceutical Research

The investigation of the transcription factor Fli-1 as a therapeutic strategy has witnessed a surge in recent years. In 2012, Y-J Li, et al. conducted a screening of six drug classes capable of inhibiting Fli-1 activity: cardenolides, calcium ionophores, topoisomerase I inhibitors, protein synthesis inhibitors, chemotherapeutic drugs, and others [64]. Subsequent verification has been performed for some of these compounds in in vitro and in vivo studies. Moreover, there exists contemporary literature highlighting Fli-1 as a promising therapeutic target [65]. The tumor cells in patients with Ewing’s sarcoma also harbor a fusion gene resulting from the fusion of two distinct genes. This genetic alteration leads to the production of a chimeric protein known as EWS-FLI1, which exerts profound oncogenic effects. EWS-FLI1 modulates the expression of numerous genes, making it an attractive therapeutic target in the field of oncology. Given that Fli-1 exhibits bidirectional regulation on target genes, current research also encompasses the study of activators alongside inhibitors targeting Fli-1. Table 4 provides an overview of drug studies focusing on Fli-1. With the emergence of novel technologies, it is anticipated that disease treatments will witness the development of Fli-1 inhibitors in the future.

## 5. Conclusions

The reviewed evidence underscores Fli-1 as a pivotal regulator of inflammation that has a key role in modulating inflammatory mediator expression and immune cell migration. Despite its potential as a therapeutic target, the complexities of Fli-1′s interactions, including its interplay with other proteins, transcription factors, and post-translational modifications, remain understudied. Promising data, however, highlight Fli-1 inhibitors as effective in ameliorating lupus nephritis in preclinical models, suggesting its therapeutic potential for inflammatory and autoimmune diseases. As research progresses, further exploration of Fli-1′s regulatory mechanisms and therapeutic applications could unlock new avenues for addressing inflammatory and autoimmune disorders.

## Figures and Tables

**Table 1:** Summary of interleukins regulated by Fli-1.

Interleukin	Stimuli	Cell Types In Vitro	Regulation In Vitro *	Regulation Through Binding to Its Promoter	Other Mechanism	Animal Model	Tissue/Cells In Vivo	Regulation In Vivo *	Ref.
IL-1β	Unknown	Unknown	Unknown	Unknown	Unknown	Bleomycin-induced SSc	Skin	Negative	[23]
						MRL/lpr mice	Kidney	Positive	[14]
						CLP-induced sepsis *	Lung pericytes	Positive	[19]
IL-4	Unknown	Unknown	Unknown	Unknown	Unknown	Bleomycin-induced SSc *	Skin	Negative	[23]
						MRL/lpr mice	Splenic T cells	Positive	[21]
IL-6	LPS	Lung pericytes	Positive	Fli-1 binding to the IL-6 promoter	Fli-1 drives transcription from the IL-6 promoter	Bleomycin-induced SSc	Skin	Negative	[19,23,24]
	LPS	Mouse endothelial cells MS1	Positive			MRL/lpr mice	Serum, kidney and splenic T cells	Positive	[11,14]
IL-10	LPS with or without IFNγ	Primary human monocytes	Positive	Unknown	Unknown	Bleomycin-induced SSc	Skin	Negative	[23,25]
IL-12a	Unknown	Unknown	Unknown	Unknown	Unknown	Bleomycin-induced SSc	Skin	Positive	[23]
						MRL/lpr mice	Splenic B cells	Negative	[22]
IL-17A	Unknown	Unknown	Unknown	Unknown	Unknown	Bleomycin-induced SSc	Skin	Negative	[23]
						MRL/lpr mice	Kidney	Positive	[14]
IL-18	Bacterial outer membrane vesicles (contain LPS)	Mouse lung pericytes	Positive	Unknown	Unknown	MRL/lpr mice	Kidney	Positive	[14,20]
						CLP-induced sepsis	Lung pericytes	Positive	[19]
IL-27	LPS	Mouse peritoneal macrophages, mouse fibroblast L929	Positive	Directly binds to the IL-27 promoter in mouse peritoneal macrophages	Fli-1 increases IL-27 p28 promoter-controlled gene transcription and cooperates with IRF1 to regulate IL-27 p28 gene expression	Unknown	Unknown	Unknown	[26]
IL-33	Unknown	Dermal fibroblasts	Negative	Unknown	Both of IL-1β and TNFα induced the dissociation of Fli1 from the IL-33 promoter	Bleomycin-induced SSc	Skin	Negative	[24]

* CLP: Cecal ligation and puncture; SSc: Systemic sclerosis; Positive/Negative: Indicate whether the expression or activation of Fli-1 positively or negatively regulates interleukin gene expression in cells or animal models.

**Table 2:** Summary of chemokines regulated by Fli-1.

Chemokine	Stimuli	Cell Types In Vitro	Regulation In Vitro ^a^	Regulation Through Binding to Its Promoter	Other Mechanism	Animal Model	Tissue/Cells In Vivo	Regulation In Vivo ^a^	Ref.
CCL2 (MCP-1)	LPS	Lung pericytes, primary endothelial cells from Fli-1 +/− NZM2410 mice and MS1 endothelial cells	Positive	Fli-1 binding to the MCP-1 promoter. Fli-1 drives transcription from the MCP-1 promoter.	Mutation of the Fli-1 DNA binding domain partially inhibits transcriptional activation from the MCP-1 promoter. Fli-1 interacts with the Ets-1 transcription factor to drive transcription from the MCP-1 promoter. Fli-1 and NFκB p65 enhance transcription from the MCP-1 promoter, while NFκB p50 and Sp1 suppress it. Ets-1 binding sites located in the distal and proximal promoter region are important for Fli-1 transcriptional activation.	Bleomycin-induced SSc ^a^	Skin	Negative	[8,23]
						MRL/lpr mice	kidneys	Positive	[19,30]
						NZM2410 mice	Kidney, serum	Positive	[9]
CCL3	Unknown	Unknown	Unknown	Unknown	Unknown	MRL/lpr mice	kidneys	Positive	[30]
CCL4	Unknown	Unknown	Unknown	Unknown	Unknown	MRL/lpr mice	kidneys	Positive	[30]
CCL5 (RANTES)	LPS	Mouse endothelial cells MS1	Positive	Fli-1 binds to the CCL5 promoter	Fli-1 drives transcription from the CCL5 promoter in a dose-dependent fashion. Ets1 acts as a dominant negative transcription factor to Fli-1 in the context of the CCL5 promoter. Activation of the CCL5 promoter by Fli-1 occurs between −746 bp and −520 bp. Fli-1 drives transcription from the CCL5 promoter more strongly than Ets1. Fli-1 regulates CCL5 through direct binding of the promoter.	MRL/lpr mice	kidneys	Positive	[30]
						NZM2410 mice	kidneys	Positive	[10]
CXCL2	LPS or TNFα	Mouse endothelial cells MS1 and HUVECs	Positive	Fli-1 binding to the CXCL2 promoter	Drives transcription from the CXCL2 promoter, Fli-1 regulates CXCL2 expression by directly binding to the promoter. NFκB acts in an additive manner.	Unknown	Unknown	Unknown	[13]
CXCL5	None	HDMECs	Positive	Unknown	Unknown	Fli-1 ECKO mice ^a^	Skin (dermal small vessels)	Positive	[32]
CXCL6	LPS	Human dermal fibroblasts and HDMEC,	Negative	Unknown	Unknown	Unknown	Unknown	Unknown	[33]
		peritoneal macrophages from Fli1+/− mice	Positive	Unknown	Unknown	Unknown	Unknown	Unknown	[33]
CXCL9	Unknown	Unknown	Unknown	Unknown	Unknown	MRL/lpr	Kidney	Positive	[31]
CXCL10	Unknown	HUVECs, human renal glomerular endothelial cells (HRGECs), and mouse endothelial MS1 cells	Positive	FLI-1 binds to the Cxcl10 promoter but failed to directly drive transcription from the human CXCL10 promoter	The DNA-binding domain of FLI-1 is necessary for its regulation of CXCR3 promoter activity in T cells	MRL/lpr	Kidney	Positive	[31,34]
CXCL13	LPS	Peritoneal macrophages from Fli1+/− mice	Negative	Unknown	Unknown	Unknown	Unknown	Unknown	[35]

**^a^** SSc, Systemic sclerosis; Fli-1 ECKO mice: mice with Fli-1 knocked out in endothelial cells; Positive/Negative: Indicate whether the expression or activation of Fli-1 positively or negatively regulates chemokine gene expression in cells or animal models.

**Table 3:** Summary of other inflammatory mediators regulated by Fli-1.

Inflammatory Mediators	Stimuli	Cell Types In Vitro	Regulation In Vitro ^a^	Regulation Through Binding to Its Promoter	Other Mechanism	Animal Model	Tissue/Cells In Vivo	Regulation In Vivo ^a^	Ref.
G-CSF	LPS	Lung pericytes, MS1 mouse endothelial cells, and HUVECs	Positive	Fli-1 binds to the proximal region of the G-CSF promoter. Fli-1 drives transcription from the G-CSF promoter.	Fli-1 regulates G-CSF expression by directly binding to the promoter. The role of acetylation in Fli-1 driven activation of the G-CSF promoter.	Unknown	Unknown	Unknown	[12,19]
GM-CSF	LPS, TNFα, IFNγ	T cells and HUVECs	Positive	Fli-1 binds directly to the GM-CSF promoter	Mutation of a known phosphorylation site within the Fli-1 protein led to a significant increase in GM-CSF promoter activation.	Unknown	T cells	Positive	[36]
MMP (MMP1, MMP3, MMP10)	LPS with or without IFNγ	Primary human monocytes	Positive	Unknown	Unknown	Unknown	Unknown	Unknown	[25]
MMP1	/	Normal dermal fibroblasts	Positive	Unknown	Unknown	Unknown	Unknown	Unknown	[37]
Caspase-1	OMV (contain LPS)	Mouse lung pericytes	Positive	Binding to its promoter	Fli-1 drives transcription from the caspase-1 promoter.	CLP-induced sepsis mice	Lung pericytes	Positive	[19,20]
Platelet factor 4 (PF4)	FLI-1 vector	HepG2 cells	Positive	FLI-1, ELF-1, and GABP bind to the −51 ETS site. FLI-1, ELF-1, and GABP activate the PF4 promoter through the −51 ETS site.	FLI-1 and GATA-1 synergistically activate the PF4 promoter. FLI-1 activates the PF4 promoter through the −51 ETS site.	Unknown	Unknown	Unknown	[38]
Chemerin	NA	HDMEC	Negative	Unknown	Unknown	Bleomycin-induced SSc ^a^	Skin	Negative	[39]
TNFα	Unknown	Unknown	Unknown	Unknown	Unknown	Bleomycin-induced SSc	Skin	Negative	[23]
Caspase-11	Unknown	Unknown	Unknown	Unknown	Unknown	CLP-induced sepsis mice ^a^	Lung pericytes	Positive	[19]
IFNγ	Unknown	Unknown	Unknown	Unknown	Unknown	Bleomycin-induced SSc	Skin	Negative	[23]
Vascular endothelial growth factor (VEGF)	LPS	Lung pericytes	Positive	Unknown	Unknown	Unknown	Unknown	Unknown	[19]
Keratinocyte chemoattractant	LPS	Lung pericytes	Positive	Unknown	Unknown	Unknown	Unknown	Unknown	[19]
Flt3L (Fms-like tyrosine kinase 3 ligand)	Flt3L, stem cell factor, IL-6, IL-6R, long-range insulin-like growth factor-1.	Multipotent progenitors (MPPs) from Fli-1∆CTA/∆CTA B6 mice	Positive	Fli-1 binding to the Flt3L promoter.	Unknown	Unknown	Unknown	Unknown	[7]
Single immunoglobulin IL-1 related receptor (SIGIRR)	Ewing tumor cells	EWSR1-FLI1 fusion reduction	Unknown	Unknown	Interacting with the TGFBR2 promoter to suppress transcriptional activity.	Unknown	Unknown	Unknown	[40]

**^a^** SSc: Systemic sclerosis; CLP: Cecal ligation and puncture; Positive/Negative: Indicate whether the expression or activation of Fli-1 positively or negatively regulates gene expression in cells or animal models.

**Table 4:** Fli-1 as a potential target for intervention.

Drug	Disease
Suppression of Fli-1	
Antisense oligonucleotide Fli-1 Gapmer	Alzheimer’s disease [66]
Calcimycin	Leukemia [64]
Camptothecin, topotecan, and etoposide	Graft-versus-host disease [41], lupus nephritis [67], and hematologic tumors [64]
A665, A661, A1544, and A1545	Hematologic tumors [68,69]
YK-4-279	Vascular proliferative disorders and tumors [70,71]
Lumefantrine	Glioblastomae [72]
Activation of Fli-1	
Bosentan, ciprofloxacin, and cyclophosphamide	Scleroderma [73,74,75]
Phorbol ester-like compounds	Hematologic tumors [76]

## Data Availability

Not applicable.

## References

[1] Heidland A., Klassen A., Rutkowski P., Bahner U. (2006). The contribution of Rudolf Virchow to the concept of inflammation: What is still of importance?. J. Nephrol..

[2] Orozco L.D., Bennett B.J., Farber C.R., Ghazalpour A., Pan C., Che N., Wen P., Qi H.X., Mutukulu A., Siemers N. (2012). Unraveling Inflammatory Responses using Systems Genetics and Gene-Environment Interactions in Macrophages. Cell.

[3] Ben-David Y., Giddens E.B., Bernstein A. (1990). Identification and mapping of a common proviral integration site Fli-1 in erythroleukemia cells induced by Friend murine leukemia virus. Proc. Natl. Acad. Sci. USA.

[4] Li Y., Luo H., Liu T., Zacksenhaus E., Ben-David Y. (2015). The ets transcription factor Fli-1 in development, cancer and disease. Oncogene.

[5] Truong A.H., Ben-David Y. (2000). The role of Fli-1 in normal cell function and malignant transformation. Oncogene.

[6] Watson D.K., E Smyth F., Thompson D.M., Cheng J.Q., Testa J.R., Papas T.S., Seth A. (1992). The ERGB/Fli-1 gene: Isolation and characterization of a new member of the family of human ETS transcription factors. Cell Growth Differ..

[7] Suzuki E., Williams S., Sato S., Gilkeson G., Watson D.K., Zhang X.K. (2013). The transcription factor Fli-1 regulates monocyte, macrophage and dendritic cell development in mice. Immunology.

[8] Richard M.L.L., Nowling T.K., Brandon D., Watson D.K., Zhang X.K. (2015). Fli-1 controls transcription from the MCP-1 gene promoter, which may provide a novel mechanism for chemokine and cytokine activation. Mol. Immunol..

[9] Suzuki E., Karam E., Williams S., Watson D.K., Gilkeson G., Zhang X.K. (2012). Fli-1 transcription factor affects glomerulonephritis development by regulating expression of monocyte chemoattractant protein-1 in endothelial cells in the kidney. Clin. Immunol..

[10] Lennard R.M., Sato S., Suzuki E., Williams S., Nowling T.K., Zhang X.K. (2014). The Fli-1 transcription factor regulates the expression of CCL5/RANTES. J. Immunol..

[11] Sato S., Richard M.L., Brandon D., Buie J.N.J., Oates J.C., Gilkeson G.S., Zhang X.K. (2014). A Critical Role of the Transcription Factor Fli-1 in Murine Lupus Development by Regulation of Interleukin-6 Expression. Arthritis Rheumatol..

[12] Lennard Richard M.L., Brandon D., Lou N., Sato S., Caldwell T., Nowling T.K., Gilkeson G., Zhang X.K. (2016). Acetylation impacts Fli-1-driven regulation of granulocyte colony stimulating factor. Eur. J. Immunol..

[13] Lou N., Richard M.L.L., Yu J., Kindy M., Zhang X.K. (2017). The Fli-1 transcription factor is a critical regulator for controlling the expression of chemokine C-X-C motif ligand 2 (CXCL2). Mol. Immunol..

[14] Sato S., Zhang X.K., Temmoku J., Fujita Y., Matsuoka N., Yashiro-Furuya M., Asano T., Kobayashi H., Watanabe H., Migita K. (2020). Ets Family Transcription Factor Fli-1 Promotes Leukocyte Recruitment and Production of IL-17A in the MRL/Lpr Mouse Model of Lupus Nephritis. Cells.

[15] Cui J.-W., Vecchiarelli-Federico L.M., Li Y.-J., Wang G.-J., Ben-David Y. (2009). Continuous Fli-1 expression plays an essential role in the proliferation and survival of F-MuLV-induced erythroleukemia and human erythroleukemia. Leukemia.

[16] Kovar H. (2014). Blocking the road, stopping the engine or killing the driver? Advances in targeting EWS/FLI-1 fusion in Ewing sarcoma as novel therapy. Expert Opin. Ther. Targets.

[17] Ichimura Y., Asano Y., Akamata K., Noda S., Taniguchi T., Takahashi T., Toyama T., Tada Y., Sugaya M., Sato S. (2015). Progranulin Overproduction Due to Fli-1 Deficiency Contributes to the Resistance of Dermal Fibroblasts to Tumor Necrosis Factor in Systemic Sclerosis. Arthritis Rheumatol..

[18] Noda S., Asano Y., Nishimura S., Taniguchi T., Fujiu K., Manabe I., Nakamura K., Yamashita T., Saigusa R., Akamata K. (2014). Simultaneous downregulation of KLF5 and Fli1 is a key feature underlying systemic sclerosis. Nat. Commun..

[19] Li P., Zhou Y., Goodwin A.J., A Cook J., Halushka P.V., Zhang X.K., Wilson C.L., Schnapp L.M., Zingarelli B., Fan H. (2018). Fli-1 Governs Pericyte Dysfunction in a Murine Model of Sepsis. J. Infect. Dis..

[20] Li P., Goodwin A.J., Cook J.A., Halushka P.V., Zhang X.K., Fan H. (2019). Fli-1 transcription factor regulates the expression of caspase-1 in lung pericytes. Mol. Immunol..

[21] Richard E.M., Thiyagarajan T., Bunni M.A., Basher F., Roddy P.O., Siskind L.J., Nietert P.J., Nowling T.K. (2013). Reducing FLI1 Levels in the MRL/lpr Lupus Mouse Model Impacts T Cell Function by Modulating Glycosphingolipid Metabolism. PLoS ONE.

[22] Bradshaw S., Zheng W.J., Tsoi L.C., Gilkeson G., Zhang X.K. (2008). A role for Fli-1 in B cell proliferation: Implications for SLE pathogenesis. Clin. Immunol..

[23] Taniguchi T., Asano Y., Akamata K., Noda S., Takahashi T., Ichimura Y., Toyama T., Trojanowska M., Sato S. (2015). Fibrosis, vascular activation, and immune abnormalities resembling systemic sclerosis in bleomycin-treated Fli-1-haploinsufficient mice. Arthritis Rheumatol..

[24] Saigusa R., Asano Y., Taniguchi T., Hirabayashi M., Nakamura K., Miura S., Yamashita T., Takahashi T., Ichimura Y., Toyama T. (2018). Fli1-haploinsufficient dermal fibroblasts promote skin-localized transdifferentiation of Th2-like regulatory T cells. Arthritis Res. Ther..

[25] Ho H.H., Ivashkiv L.B. (2010). Downregulation of Friend Leukemia Virus Integration 1 as a Feedback Mechanism That Restrains Lipopolysaccharide Induction of Matrix Metalloproteases and Interleukin-10 in Human Macrophages. J. Interf. Cytokine Res..

[26] Gao P., Yuan M., Ma X., Jiang W., Zhu L., Wen M., Xu J., Liu Q., An H. (2016). Transcription factor Fli-1 positively regulates lipopolysaccharide-induced interleukin-27 production in macrophages. Mol. Immunol..

[27] Khan M.A., Khurana N., Ahmed R.S., Umar S., Sarwar A.H.M.G., Alam Q., Kamal M.A., Ashraf G.M. (2019). Chemokines: A Potential Therapeutic Target to Suppress Autoimmune Arthritis. Curr. Pharm. Des..

[28] Viola A., Luster A.D. (2008). Chemokines and Their Receptors: Drug Targets in Immunity and Inflammation. Annu. Rev. Pharmacol. Toxicol..

[29] Chen K., Bao Z., Tang P., Gong W., Yoshimura T., Wang J.M. (2018). Chemokines in homeostasis and diseases. Cell Mol. Immunol..

[30] Sato S., Zhang X.K. (2014). The Friend leukaemia virus integration 1 (Fli-1) transcription factor affects lupus nephritis development by regulating inflammatory cell infiltration into the kidney. Clin. Exp. Immunol..

[31] Sundararaj K.P., Thiyagarajan T., Molano I., Basher F., Powers T.W., Drake R.R., Nowling T.K. (2015). FLI1 Levels Impact CXCR3 Expression and Renal Infiltration of T Cells and Renal Glycosphingolipid Metabolism in the MRL/lpr Lupus Mouse Strain. J. Immunol..

[32] Ichimura Y., Asano Y., Akamata K., Takahashi T., Noda S., Taniguchi T., Toyama T., Aozasa N., Sumida H., Kuwano Y. (2014). Fli1 deficiency contributes to the suppression of endothelial CXCL5 expression in systemic sclerosis. Arch. Dermatol. Res..

[33] Taniguchi T., Asano Y., Nakamura K., Yamashita T., Saigusa R., Ichimura Y., Takahashi T., Toyama T., Yoshizaki A., Sato S. (2017). Fli1 Deficiency Induces CXCL6 Expression in Dermal Fibroblasts and Endothelial Cells, Contributing to the Development of Fibrosis and Vasculopathy in Systemic Sclerosis. J. Rheumatol..

[34] Wang X., Richard M.L., Caldwell T.S., Sundararaj K., Sato S., Nowling T.K., Zhang X.K. (2023). Role of the transcription factor Fli-1 on the CXCL10/CXCR3 Axis. Front. Immunol..

[35] Taniguchi T., Miyagawa T., Toyama S., Yamashita T., Nakamura K., Saigusa R., Ichimura Y., Takahashi T., Toyama T., Yoshizaki A. (2018). CXCL13 produced by macrophages due to Fli1 deficiency may contribute to the development of tissue fibrosis, vasculopathy and immune activation in systemic sclerosis. Exp. Dermatol..

[36] Wang X., Richard M.L., Li P., Henry B., Schutt S., Yu X.-Z., Fan H., Zhang W., Gilkeson G., Zhang X.K. (2021). Expression of GM-CSF Is Regulated by Fli-1 Transcription Factor, a Potential Drug Target. J. Immunol..

[37] Nakerakanti S.S., Kapanadze B., Yamasaki M., Markiewicz M., Trojanowska M. (2006). Fli1 and Ets1 Have Distinct Roles in Connective Tissue Growth Factor/CCN2 Gene Regulation and Induction of the Profibrotic Gene Program. J. Biol. Chem..

[38] Okada Y., Nobori H., Shimizu M., Watanabe M., Yonekura M., Nakai T., Kamikawa Y., Wakimura A., Funahashi N., Naruse H. (2011). Multiple ETS Family Proteins Regulate PF4 Gene Expression by Binding to the Same ETS Binding Site. PLoS ONE.

[39] Akamata K., Asano Y., Taniguchi T., Yamashita T., Saigusa R., Nakamura K., Noda S., Aozasa N., Toyama T., Takahashi T. (2015). Increased expression of chemerin in endothelial cells due to Fli1 deficiency may contribute to the development of digital ulcers in systemic sclerosis. Rheumatology.

[40] Hahm K.-B., Cho K., Lee C., Im Y.-H., Chang J., Choi S.-G., Sorensen P.H., Thiele C.J., Kim S.-J. (1999). Repression of the gene encoding the TGF-beta type II receptor is a major target of the EWS-FLI1 oncoprotein. Nat. Genet..

[41] Schutt S.D., Wu Y., Kharel A., Bastian D., Choi H.-J., Sofi M.H., Mealer C., Mims B.M., Nguyen H., Liu C. (2022). The druggable transcription factor Fli-1 regulates T cell immunity and tolerance in graft-versus-host disease. J. Clin. Investig..

[42] Mathenia J., Reyes-Cortes E., Williams S., Molano I., Ruiz P., Watson D.K., Gilkeson G.S., Zhang X.K. (2010). Impact of Fli-1 transcription factor on autoantibody and lupus nephritis in NZM2410 mice. Clin. Exp. Immunol..

[43] Yan X., Yu Y., Li L., Chen N., Song W., He H., Dong J., Liu X., Cui J. (2018). Friend leukemia virus integration 1 is a predictor of poor prognosis of breast cancer and promotes metastasis and cancer stem cell properties of breast cancer cells. Cancer Med..

[44] Hollenhorst P.C., Jones D.A., Graves B.J. (2004). Expression profiles frame the promoter specificity dilemma of the ETS family of transcription factors. Nucleic Acids Res..

[45] Scholz R., Brosamle D., Yuan X., Beyer M., Neher J.J. (2024). Epigenetic control of microglial immune responses. Immunol. Rev..

[46] Jackers P., Szalai G., Moussa O., Watson D.K. (2004). Ets-dependent Regulation of Target Gene Expression during Megakaryopoiesis. J. Biol. Chem..

[47] Asano Y., Trojanowska M. (2009). Phosphorylation of Fli1 at threonine 312 by protein kinase C delta promotes its interaction with p300/CREB-binding protein-associated factor and subsequent acetylation in response to transforming growth factor beta. Mol. Cell. Biol..

[48] Chen Z., Arai E., Khan O., Zhang Z., Ngiow S.F., He Y., Huang H., Manne S., Cao Z., Baxter A.E. (2021). In vivo CD8(+) T cell CRISPR screening reveals control by Fli1 in infection and cancer. Cell.

[49] Georgiou P., Maroulakou I., Green J., Dantis P., Romano-Spica V., Kottaridis S., Lautenberger J., Watson D., Papas T., Fischinger P. (1996). Expression of ets family of genes in systemic lupus erythematosus and Sjogren’s syndrome. Int. J. Oncol..

[50] Zhang X.K., Gallant S., Molano I., Moussa O.M., Ruiz P., Spyropoulos D.D., Watson D.K., Gilkeson G. (2004). Decreased Expression of the Ets Family Transcription Factor Fli-1 Markedly Prolongs Survival and Significantly Reduces Renal Disease in MRL/*lpr* Mice. J. Immunol..

[51] Kubo M., Czuwara-Ladykowska J., Moussa O., Markiewicz M., Smith E., Silver R.M., Jablonska S., Blaszczyk M., Watson D.K., Trojanowska M. (2003). Persistent Down-Regulation of Fli1, a Suppressor of Collagen Transcription, in Fibrotic Scleroderma Skin. Am. J. Pathol..

[52] Wang Y., Fan P., Kahaleh B. (2006). Association between enhanced type I collagen expression and epigenetic repression of the *FLI1* gene in scleroderma fibroblasts. Arthritis Rheum..

[53] Asano Y., Bujor A.M., Trojanowska M. (2010). The impact of Fli1 deficiency on the pathogenesis of systemic sclerosis. J. Dermatol. Sci..

[54] Singh N., Baby D., Rajguru J.P., Patil P.B., Thakkannavar S.S., Pujari V.B. (2019). Inflammation and cancer. Ann. Afr. Med..

[55] Ben-David Y., Gajendran B., Sample K.M., Zacksenhaus E. (2022). Current insights into the role of Fli-1 in hematopoiesis and malignant transformation. Cell. Mol. Life Sci..

[56] Wang J., Wang C., Hu A., Yu K., Kuang Y., Gajendran B., Zacksenhaus E., Sample K.M., Xiao X., Liu W. (2024). FLI1 induces erythroleukemia through opposing effects on UBASH3A and UBASH3B ex-pression. BMC Cancer.

[57] Maiorino L., Egeblad M. (2019). Tumours pick the path to cancer inflammation. Nat. Cell Biol..

[58] Diakos C.I., Charles K.A., McMillan D.C., Clarke S.J. (2014). Cancer-related inflammation and treatment effectiveness. Lancet Oncol..

[59] Germano G., Allavena P., Mantovani A. (2008). Cytokines as a key component of cancer-related inflammation. Cytokine.

[60] Lattanzio G., Libert C., Aquilina M., Cappelletti M., Ciliberto G., Musiani P., Poli V. (1997). Defective development of pristane-oil-induced plasmacytomas in interleukin-6-deficient BALB/c mice. Am. J. Pathol..

[61] Garlanda C., Riva F., Veliz T., Polentarutti N., Pasqualini F., Radaelli E., Sironi M., Nebuloni M., Zorini E.O., Scanziani E. (2007). Increased susceptibility to colitis-associated cancer of mice lacking TIR8, an inhibitory member of the interleukin-1 receptor family. Cancer Res..

[62] Singer M., Deutschman C.S., Seymour C.W., Shankar-Hari M., Annane D., Bauer M., Bellomo R., Bernard G.R., Chiche J.-D., Coopersmith C.M. (2016). The Third International Consensus Definitions for Sepsis and Septic Shock (Sepsis-3). JAMA.

[63] Wu Y., Li P., Goodwin A.J., A Cook J., Halushka P.V., Zingarelli B., Fan H. (2020). miR-145a Regulates of Pericyte Dysfunction in a Murine Model of Sepsis. J. Infect. Dis..

[64] Li Y.-J., Zhao X., Vecchiarelli-Federico L.M., Datti A., Cheng Y., Ben-David Y. (2012). Drug-mediated inhibition of Fli-1 for the treatment of leukemia. Blood Cancer J..

[65] Zhang L., Ge T., Cui J. (2024). FLI-1-driven regulation of endothelial cells in human diseases. J. Transl. Med..

[66] Li P., Wu Y., Hamlett E.D., Goodwin A.J., Halushka P.V., Carroll S.L., Liu M., Fan H. (2022). Suppression of Fli-1 protects against pericyte loss and cognitive deficits in Alzheimer’s disease. Mol. Ther..

[67] Wang X., Oates J.C., Helke K.L., Gilkeson G.S., Zhang X.K. (2021). Camptothecin and Topotecan, Inhibitors of Transcription Factor Fli-1 and Topoisomerase, Markedly Ameliorate Lupus Nephritis in (NZB × NZW)F1 Mice and Re-duce the Production of Inflammatory Mediators in Human Renal Cells. Arthritis Rheumatol..

[68] Liu T., Xia L., Yao Y., Yan C., Fan Y., Gajendran B., Yang J., Li Y.-J., Chen J., Filmus J. (2019). Identification of diterpenoid compounds that interfere with Fli-1 DNA binding to suppress leukemogenesis. Cell Death Dis..

[69] Song J., Yuan C., Yang J., Liu T., Yao Y., Xiao X., Gajendran B., Xu D., Li Y., Wang C. (2018). Novel flavagline-like compounds with potent Fli-1 inhibitory activity suppress diverse types of leukemia. FEBS J..

[70] Schafer C.M., Gurley J.M., Kurylowicz K., Lin P.K., Chen W., Elliott M.H., Davis G.E., Bhatti F., Griffin C.T. (2020). An inhibitor of endothelial ETS transcription factors promotes physiologic and therapeutic vessel regression. Proc. Natl. Acad. Sci. USA.

[71] Chen E., Wu J., Huang J., Zhu W., Sun H., Wang X., Lin D., Li X., Shi D., Liu Z. (2024). FLI1 promotes IFN-gamma-induced kynurenine production to impair anti-tumor immunity. Nat. Commun..

[72] Rajesh Y., Biswas A., Kumar U., Banerjee I., Das S., Maji S., Das S.K., Emdad L., Cavenee W.K., Mandal M. (2020). Lumefantrine, an antimalarial drug, reverses radiation and temozolomide resistance in gli-oblastoma. Proc. Natl. Acad. Sci. USA.

[73] Akamata K., Asano Y., Aozasa N., Noda S., Taniguchi T., Takahashi T., Ichimura Y., Toyama T., Sato S. (2014). Bosentan reverses the pro-fibrotic phenotype of systemic sclerosis dermal fibroblasts via increasing DNA binding ability of transcription factor Fli1. Arthritis Res. Ther..

[74] Yamashita T., Asano Y., Saigusa R., Taniguchi T., Hirabayashi M., Miyagawa T., Nakamura K., Miura S., Yoshizaki A., Trojanowska M. (2019). Cyclophosphamide Pulse Therapy Normalizes Vascular Abnormalities in a Mouse Model of Systemic Sclerosis Vasculopathy. J. Investig. Dermatol..

[75] Bujor A.M., Haines P., Padilla C., Christmann R.B., Junie M., Sampaio-Barros P.D., Lafyatis R., Trojanowska M. (2012). Ciprofloxacin has antifibrotic effects in scleroderma fibroblasts via downregulation of Dnmt1 and upregulation of Fli1. Int. J. Mol. Med..

[76] Liu T., Yao Y., Zhang G., Wang Y., Deng B., Song J., Li X., Han F., Xiao X., Yang J. (2017). A screen for Fli-1 transcriptional modulators identifies PKC agonists that induce erythroid to megakaryocytic differentiation and suppress leukemogenesis. Oncotarget.

